# Differential diagnosis of benign lesions and lung adenocarcinoma presenting as lung-RADS 2022 category 4B solid nodules based on multiscale CT radiomics

**DOI:** 10.1186/s12885-026-15883-w

**Published:** 2026-03-22

**Authors:** Jiayue Xie, Siyu Che, Junjie Li, Yuxin Niu, Yifan He, Shuai Hu, Dongxue Qin, Zhiyong Li

**Affiliations:** 1 Department of Radiology, the First Affiliated Hospital of Dalian Medical University, Zhongshan road No.222, Xigang District, Dalian, Liaoning Province 116011 China; 2 Department of Radiology, the Second Hospital of Dalian Medical University, Dalian, Liaoning Province China

**Keywords:** Radiomics, Contrast enhanced, Lung adenocarcinoma, Pulmonary nodule, Differential diagnosis

## Abstract

**Purpose:**

Based on multiscale computed tomography (CT) radiomics, a better model was established to differentially diagnose benign lesions and lung adenocarcinoma of Lung-RADS 2022 category 4B solid lung nodules.

**Materials and methods:**

The retrospective study included 178 patients with solid pulmonary nodules were assigned to the training dataset (*n* = 124) and the testing dataset (*n* = 54). Gradient boosting decision tree (GBDT) was used to reduce the dimensionality of data and select the best radiomics features. Four models were developed by logistic regression method, namely the clinical and imaging model (CIM), the plain CT radiomics model (PRM), the enhanced CT radiomics model (ERM), and the combined model (CM). Area under the curve (AUC) evaluates the model performance. Net reclassification improvement (NRI) and the integrated discrimination index (IDI) were calculated to compare the performance of different models to determine the best model.

**Results:**

In the training dataset, the AUC of CIM, PRM, ERM, and CM were 0.795, 0.791, 0.828, and 0.888. The continuous NRI and IDI of CM was better than that of CIM, PRM, ERM (*P* < 0.001), CM is optimal. In the testing dataset, the AUC of CIM, PRM, ERM and CM were 0.810, 0.689, 0.864 and 0.881. The continuous NRI of CM was better than that of CIM, PRM, ERM (*P* < 0.050). The IDI of CM was better than that of CIM and PRM (*P* < 0.050). The AUC values of the Mayo Clinic (Mayo) model, Veterans Administration (VA) model, Peking University People’s Hospital (PKUPH) model and United Imaging Artificial Intelligence (UI AI) model were 0.419, 0.410, 0.676 and 0.675, CM is still optimal.

**Conclusion:**

Radiomics can be used as a non-invasive tool to distinguish between benign lesions and lung adenocarcinoma of Lung-RADS 2022 category 4B solid lung nodules, and CM is the best predictive model.

**Supplementary Information:**

The online version contains supplementary material available at 10.1186/s12885-026-15883-w.

## Introduction

Pulmonary nodules are one of the common basic imaging findings in many lung diseases. For the large solid nodules, there is a greater possibility of malignancy, early metastasis, and a poor prognosis [1]. Correct stratification of pulmonary nodules is more challenging in clinical work. Therefore, many relevant guidelines and consensus have been developed to support scientific management and clinical decision-making, among which, considering the diameter size of pulmonary nodules as a key risk factor [2, 3]. The American College of Radiology Lung-RADS 2022 screening criteria classified solid nodules of mean diameter greater than or equal to 8 mm as high risk (category 4) [4], solid nodules between 8 mm and 15 mm were classified as a high-risk level of category 4 A, and follow-up observation within 3 months or further detailed examination was recommended, greater than or equal to 15 mm solid nodules are classified as a high risk of category 4B, and positron emission tomography-computed tomography (PET-CT) examination, pathological tissue biopsy are directly recommended. Lung adenocarcinoma is also one of the most common types of peripheral lung cancer. Whether solid lung nodules are benign lesions or lung adenocarcinoma is a problem worthy of attention. Non-invasive biomarkers may help in identifying the population at greatest risk [5, 6]. Accurate differentiation of benign lesions of solid lung nodules and lung adenocarcinoma plays a crucial role in developing effective treatment plans and improving the quality of life of patients.

As an emerging and promising research method, imaging radiomics can quickly extract massive features from tomographic images with the help of high-throughput computing technology [7, 8]. These features are usually classified as first-level statistical features, morphological features as well as texture statistical features. Large amounts of invisible, high-dimensional, valuable data are hidden on computed tomography (CT) images. Imaging radiomics is a promising tool to identify benign and malignant lung nodules [9, 10]. The plain CT radiomics helps to distinguish between benign and malignant solid pulmonary nodules [11, 12].

Enhanced CT images usually reflect the blood supply of the lesion, and enhanced attenuation is more helpful in distinguishing benign and malignant lung lesions. Enhanced CT scan can detect lesions that are difficult to detect on plain CT scan and can accurately determine the blood supply status of the lesion. As the iodine concentration in the blood increases, the iodine concentration in different organs and diseased tissues will vary, resulting in a difference in density, this density difference helps to more accurately detect the change in hemodynamics within the lesion with the spatial heterogeneity, and further improve the accuracy of the differential diagnosis. Previous studies of studies have confirmed that enhanced CT is helpful for benign and malignant differentiation of solid lung nodules [13–16]. The research direction of enhanced CT radiomics has been making breakthroughs recently. The results of Qin Liu et al. showed that the imaging radiomics nomogram constructed based on enhanced CT can be used to predict lung adenocarcinoma [17]. Another study has also found that enhanced CT imaging can help distinguish tuberculosis and lung adenocarcinoma [18].

In this study, imaging radiomics predictive models for the diagnosis of benign lesions and lung adenocarcinoma lesions of Lung-RADS 2022 category 4B solid lung nodules were constructed to guide clinical decision-making.

## Materials and methods

### Patients

The study was approved by the ethics committees and informed consent was not required given the retrospective nature of the study.The ethical approval number is PJ-KS-KY-2021-291. With informed consent waived due to the feasibility of retrospective analysis of archived data and data de-identification. Data confidentiality was fully managed throughout the process: private information was deleted during collection, retaining only anonymous IDs and essential metrics; data was stored on physically isolated dedicated servers within the hospital and retained for 5 years before permanent destruction. All procedures complied with the Ethical Review Measures for Human-Involved Biomedical Research and the Helsinki Declaration.

This study is a retrospective study including 188 patients with Lung-RADS 2022 4B solid lung nodules from three different medical centers, including lesions from hospital 1 between January 2010 and July 2024, lesions from hospital 2 between January 2019 and January 2023, and lesions from hospital 3 between December 2017 and July 2021. All patients underwent preoperative 3 months chest CT examination.

We searched the patients for the following inclusion criteria: (a) clinical outcome(After treatment and follow-up) or sputum examination or pathologically confirmed benign nodules; pathologically confirmed malignant nodules; (b) the lesion in the peripheral region of the lung; (c) the plain scan CT and enhanced CT images with layer thickness ≤ 2.5 mm (d) the lesions were Lung-RADS 2022 4B solid lung nodules with a size greater than or equal to 15 mm; (e) patients with no history of lung surgery, radiotherapy or chemotherapy. Exclusion criteria for this study included (a) decline image quality due to artifacts, and (b) pulmonary hamartomas (pulmonary hamartomas were excluded due to their internal presence of fat and low enhancement).

After applying the inclusion and exclusion criteria, 178 patients (96 in lung adenocarcinoma group and 82 in benign lesions group) were included in the study. We included 124 patients in the training dataset with a ratio of 7:3 and 54 patients in the testing dataset. Each patient corresponds to one lesion.

The benign lesions of lung-RADS 2022 4B solid pulmonary nodules included six benign lesions that were clinically effective (The six benign lesions were significantly reduced or completely absorbed after anti-inflammatory treatment during the second examination. Follow-up visits at 40 days, 60 days, 76 days, and 4 years showed complete absorption of four benign lesions. After 7 days and 600 days of follow-up, more than 50% of the two benign lesions were absorbed), eight pulmonary tuberculosis confirmed by sputum examination, and 68 pathologically confirmed benign lesions. Malignant nodules were pathologically confirmed to be lung adenocarcinoma.

### CT image acquisition

Chest CT images were obtained using Optima CT660/Discovery CT750 HD/LightSpeed Plus/LightSpeed16/Revolution CT (General Electric, USA), uCT760 (United Imaging, China), and Brilliance 16P and ict256 (Philips, Netherlands), SOMATOM Drive/SOMATOM Definition (Siemens, Germany).

The patient was positioned in the supine position, and scanning was conducted from the lung apex to the lower edge of the costophrenic angle with deep inspiratory breath-holding imaging. The tube voltage was set within a range of 120–140 kV, while the tube current ranged from 140 to 630 mAs or was automatically adjusted according to the patient’s body weight. A matrix of 512 × 512 pixels was used, with both the reconstruction interval and thickness set to 1.0–2.5 mm. The contrast agent was injected at a rate of 2.5–3.0 ml/s, and a venous-phase contrast-enhanced CT scan was performed 55–60 s after contrast injection.

### Clinical and conventional imaging features of CT images

Clinical features include sex, age and smoking status.

The CT images were independently analyzed by two primary radiologists with chest imaging diagnostic experience: Jiayue Xie (6 years of experience) and Yuxin Niu (3 years of experience). Neither of them had access to the patients’ final pathological diagnosis results or medical history information. The chest CT images of each patient were reviewed by the two radiologists using RadiAnt DICOM Viewer 2021.2 (64-bit) software (Poland). In cases of disagreement between the two radiologists, a senior radiologist (Zhiyong Li, 30 years of diagnostic chest imaging experience) was consulted to determine the final diagnosis.

The CT images were observed using both lung window (window width: 1500 HU; window level: -600 HU) and mediastinal window (window width: 400 HU; window level: 40 HU). The conventional imaging features analyzed on CT images were as follows: (1) max diameter (the longest diameter at the maximum level of the axial lesion); (2) mean diameter (long and short diameters in the largest cross-section, in order to compute the mean) (3) location (lower lobe; upper and middle lobes); (4) spiculation (absent, present), (5) lobulation (absent, present); (6) cavity (absent, present) (7) pleural traction (absent, present); (8) air bronchogram (absent, present).

### Lesion segmentation and imaging radiomic features extraction

Prior to the segmentation, all images underwent standardized preprocessing, and set up the mediastinal window (window width: 400HU; window level: 40HU). Using the mediastinal window to extract the imaging radiomics features. The region of interest (ROI) of each CT image was segmented by a junior radiologist through the open-source software version 3Dslicer 5.0.3 (http://slicer.org/). T​h​​​​​e ROI was manually segmented for each patient. Prior to the feature extraction, a resampling step was first performed to uniformly adjust the voxel size to 1 mm×1 mm×1 mm. Using 3D Slicer software, a total of 107 imaging radiomics features were automatically extracted from VOI, including 14 shape features, 18 first-order statistical features and 75 texture features.

### Assess consistency

After a month of initial ROI segmentation, we randomly selected 30 out of the 178 lesions for inter-group and intra-group consistency evaluation. The evaluation of these 30 lesions was performed by radiologists A and B, respectively, and the final pathological results of these lesions were unknown at the time of the evaluation. To quantify the inter-observer and intra-observer agreement during feature extraction, the inter-group and intra-group correlation coefficient (ICC) was used as the evaluation indicator. After calculating the ICC for each feature, the ICC was > 0.750, indicating a good or excellent agreement for this feature.

### Feature selection and model construction

In plain CT images and in enhanced CT images, First, the radiomic feature data were normalized. Secondly, the imaging radiomics features in the training dataset were screened through correlation analysis. If the average correlation coefficient between a feature and all other features was higher than 0.800, the feature would be removed. Subsequently, the gradient boosting decision tree (GBDT) method was used to selection feature.

Based on the filtered feature set, we constructed four logistic regression models. We developed and compared four models, including clinical and imaging model (CIM), plain CT radiomics model (PRM), enhanced CT radiomics model (ERM) and combined model (CM). The CM is composed of the CIM, the PRM, and the ERM.

### Assessment model performance

We evaluated the performance of the four models on the training and testing datasets using receiver operator characteristic (ROC) curve analysis. For each model, we calculated the area under the ROC curve (AUC), sensitivity, specificity, and accuracy.

To fit each model to the ideal model, we used calibration curves as well as the Hosmer-Lemeshow test. The net reclassification improvement (NRI) and the integrated discrimination index (IDI) were calculated to compare the performance of the different models. In addition, a decision curve analysis (DCA) was applied to further evaluate the clinical efficacy of the model based on the net benefit and the corresponding probability thresholds.

**Fig. 1:**
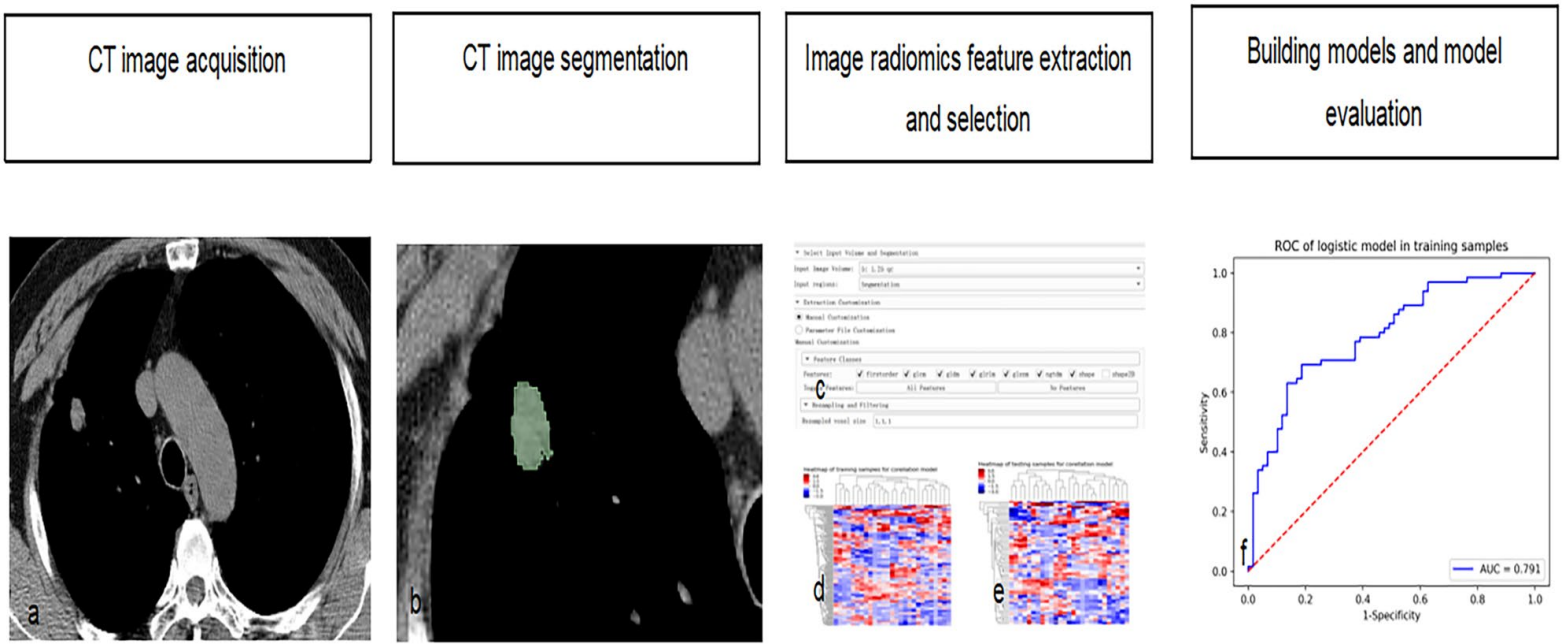
A flowchart illustrating the procedure for constructing the radiomics-based model

A flowchart illustrating the procedure for constructing the radiomics-based model in this study is shown in Fig. 1.

### Other domestic and foreign recognized lung cancer risk prediction models and United Imaging Artificial Intelligence model

In this study, three prediction models widely recognized at domestic and foreign were selected for comparative analysis. Their calculations are given in the formula (1).

1 $$\:\:Y=\frac{{e}^{x}}{{e}^{x}﹢1}$$

1) Mayo Clinic Model(Mayo) [19]: *X*=-6.8272+(0.0391×Age)+(0.7917×Cigarettes)+ (1.3388×Cancer)+(0.1274×Diameter)+(1.0407×Spiculation)+(0.7838×upper). 2) Veterans Administration model(VA) [20]: *X*=-8.404+(2.061×smoke)+(0.779×age 10)+(0.112×diameter)−(0.567×years quit 10). 3)Peking University People’s Hospital(PKUPH) model [21]: *X*=-4.496+(0.07×age)+(0.676×diameter)+(0.736×spiculation)+(1.267×family history of tumor)-(1.615×calcification)-(1.408×border). In the model mentioned above, *e* is 2.718.*Y* is the probability of malignancy in the lung nodules.

In this study, also the United Imaging Artificial Intelligence lung cancer risk prediction model (UI AI model) used in the daily work of the hospital was selected, and the image data was entered in the AI software to obtain the malignant probability value of Lung-RADS 2022 4B solid lung nodules.

This study mainly uses the testing dataset to conduct diagnostic test on the above four models (3 domestic and foreign recognized models and UI AI model), which provides a practical reference comparison for the efficacy evaluation between the CM and the above four models.

### Statistical analysis

R 4.3.2 (https://cran.r-project.org/) and IBM SPSS 26.0 software were used for the statistical analysis. Independent predictors in clinical and conventional imaging features were identified by univariate and multivariate analysis. The goodness of fit was calculated using the H-L test. The ROC analysis and the decision curve analysis was performed using R 4.3.2. The AUC, sensitivity, specificity, and accuracy were used to evaluate the model performance. The NRI and the IDI were calculated to compare the performance of the different models. NRI indicates the net proportion of correct case reclassification by the model. A positive value indicates that the new model outperforms the old model in case reclassification accuracy. IDI indicates measures the model’s overall discriminative performance. A positive IDI value indicates the new model outperforms the old model. The logistic regression coefficient method is used to explain the decision process of the machine learning model and to quantify the contribution of each feature to the prediction results.

We selected statistical methods based on the study design and outcome characteristics: (1) Logistic regression was used for model development due to its suitability for binary classification (benign vs. malignant) and interpretability of variable contributions. (2) AUC is the gold standard for evaluating model discrimination in diagnostic studies. Its value is independent of specific diagnostic thresholds, enabling comprehensive assessment of model performance.(3) NRI and IDI were applied to compare inter-model performance, as they effectively quantify incremental predictive value beyond AUC by evaluating reclassification accuracy and improvement in predicted probabilities. (4) For goodness-of-fit assessment, the Hosmer-Lemeshow (HL) test was prioritized for two key reasons: First, it is a well-established, widely accepted parametric method for evaluating the calibration of binary logistic regression models, directly quantifying the agreement between the models’ predicted probabilities and the observed event rates in the study population. Second, the HL test provides an intuitive interpretation: a P-value > 0.05 indicates no statistically significant discrepancy between predicted and observed outcomes, confirming that the models are adequately calibrated to the data. In addition to the HL test, calibration curves were also generated to visually complement the goodness-of-fit assessment, further validating the models’ fit in a clinically interpretable manner—an important supplement to the HL test’s numerical results.

For categorical variables (e.g. sex), we compared Pearson’s chi-square test with Fisher’s exact testing; For continuous variables (e.g., age) that followed the normal distribution pattern, a t-test was used for comparison. For those continuous variables that did not fit the normal distribution properties, we chose the Mann-Whitney U test for the corresponding contrast assessment. All the statistical analyses were two-tailed. A *P*-value of less than 0.050 was considered statistically significant. ICC treatment (< 0.500 poor reliability, 0.500–0.750 medium reliability, 0.750–0.900 good reliability,> 0.900 excellent reliability).

## Result

### Basic information

**Fig. 2:**
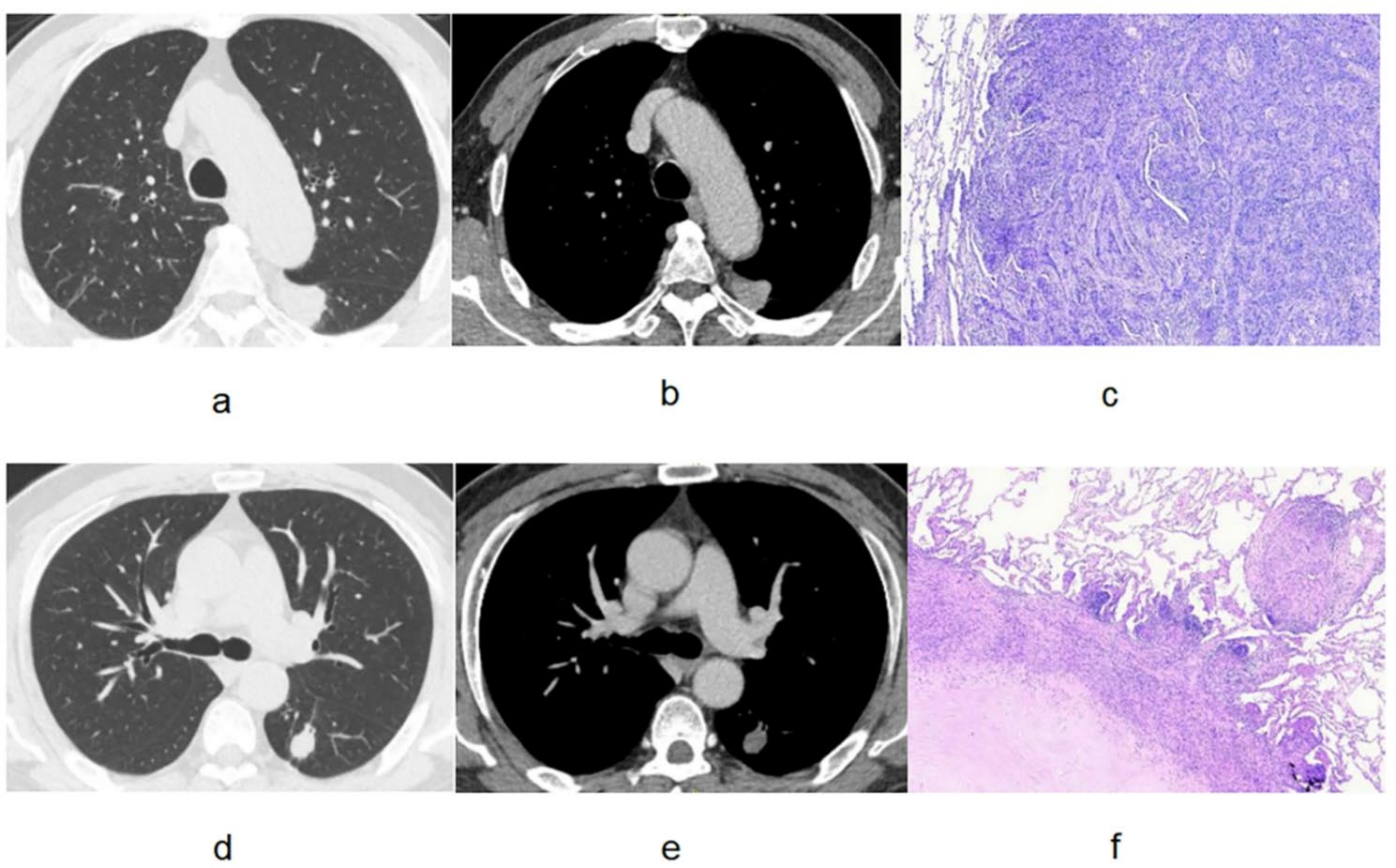
Lung-RADS 2022 category 4B solid lung nodules. **a**, **b**, and **c** A 74-year-old male patient, with the solid lung nodule with a mean diameter of 2.40 cm, was diagnosed as lung adenocarcinoma by pathological examination. **d**, **e**, and **f** A 48-year-old male patient with the solid pulmonary nodule in the lower lobe of the left lung with a mean diameter of 1.60 cm was identified as the pulmonary tuberculosis nodule by pathological diagnosis

**Fig. 3:**
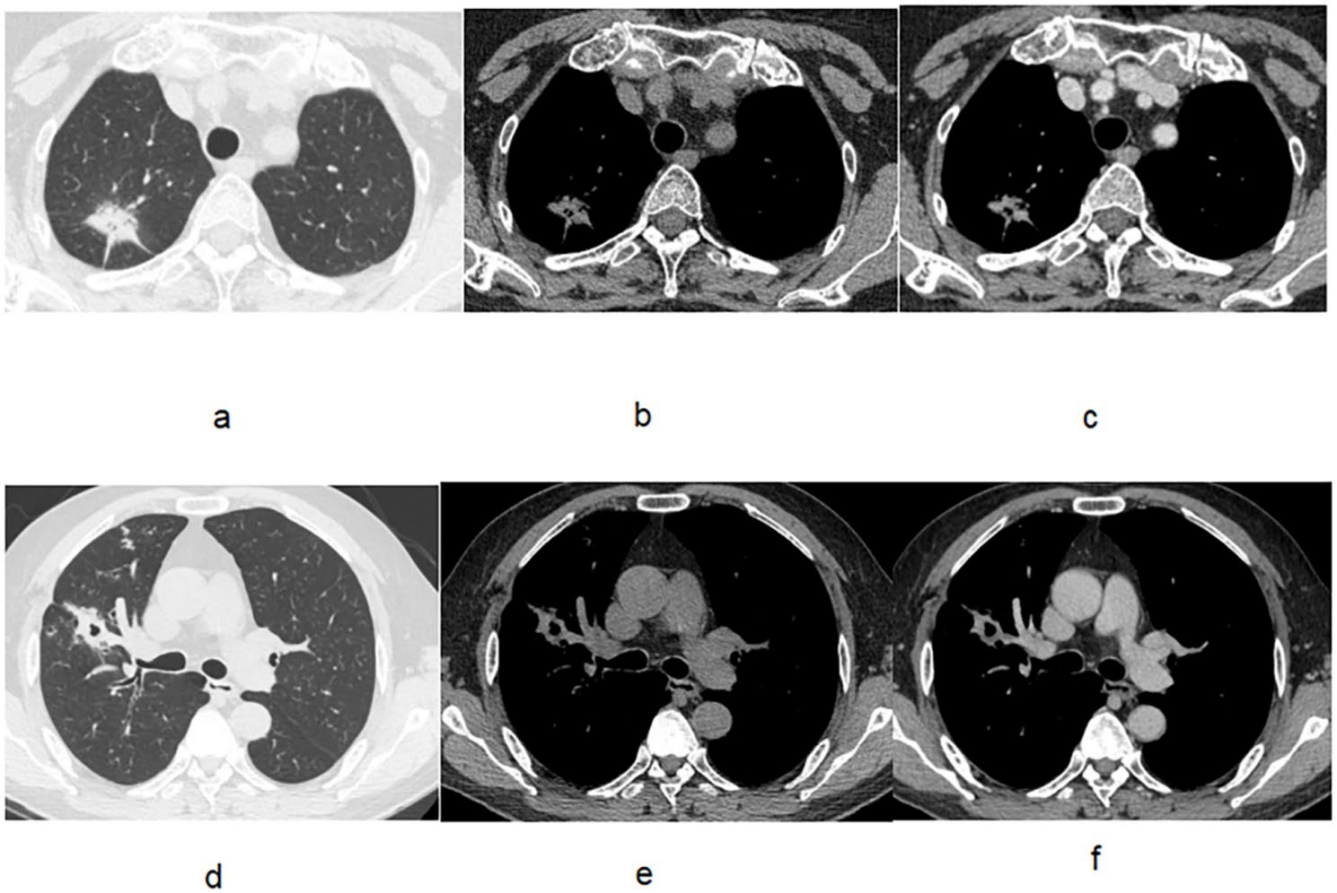
Lung-RADS 2022 category 4B solid lung nodules in lung and mediastinal windows. The Plain CT_radscore and Enhanced CT_radscore scores of malignant case for Figure (**a**, **b**, and **c**) are 1.596 and 1.987, respectively.The final calculated malignancy probability is 0.890.The Plain CT_radscore and Enhanced CT_radscore scores of benign case for the Figure (**d**, **e**, and **f**) are-2.521 and − 2.841, respectively.The final calculated malignancy probability is 0.007

The training dataset (124 patients) included 59 patients with benign lesions and 65 patients with malignant lesions. The testing dataset (54 patients) included 23 benign patients and 31 malignant patients. The mean diameter of the Lung-RADS 2022 4B solid pulmonary nodules is 1.75–2.75 cm. Supplement Fig. 1 shows the flow chart of Lung-RADS 2022 category 4B solid lung nodules selection and grouping. Examples of the lesions in the benign and lung adenocarcinoma groups are shown in Figs. 2 and 3. In the Fig. 3, for the malignant case, the Plain CT_radscore and Enhanced CT_radscore are 1.596 and 1.987, respectively, with a final calculated malignancy probability of 0.890. For the benign case, the Plain CT_radscore and Enhanced CT_radscore are − 2.521 and − 2.841, respectively, with a final calculated malignancy probability of 0.007.

In this study, 178 patients were finally included in the analysis, with a total of 96 malignant nodules, including 96 lesions of lung adenocarcinoma; A total of 82 benign nodules occurred, including 44 of inflammatory nodules, 34 of tubercular nodules, 2 of sclerosing pneumocytoma, 1 of pulmonary cryptococcosis, and 1 of pulmonary aspergillosis.

### Clinical and conventional imaging features analysis

The comparison of clinical and conventional imaging features between the benign lesion and lung adenocarcinoma groups is shown in Supplement Table 1. In the training dataset, the age of the lung adenocarcinoma group was higher than that in the benign lesion group (*P* = 0.003). Lung adenocarcinoma group had significantly more spiculation than benign lesions (*P* < 0.001).

**Table 1:** Univariable and multivariable analyses of clinical and conventional image features in the training dataset

Features	Univariable analysis			Multivariable analysis		
	OR(95% CI)		*P*	OR(95% CI)		*P*
Age	1.06	(1.02–1.09)	0.004*	1.05	(1.01–1.10)	0.013*
Sex	0.48	(0.23–0.98)	0.045*	0.35	(0.15–0.84)	0.018*
Smoking status	0.60	(0.28–1.28)	1.184			
Max diameter	0.68	(0.34–1.35)	0.274			
Mean diameter	0.98	(0.43–2.27)	0.969			
Location	1.02	(0.50–2.11)	0.953			
Spiculation	7.75	(2.89–20.74)	< 0.01*	9.49	(3.28–27.46)	< 0.001*
Lobulation	1.87	(0.77–4.58)	0.168			
Cavity	0.40	(0.14–1.14)	0.086	0.31	(0.10-1.00)	0.049*
Pleural traction	1.28	(0.46–3.58)	0.634			
Air bronchogram	1.11	(0.54–2.28)	0.772			

*OR* odds ratio, *CI* confidence interval

*P value < 0.050

In the training dataset, univariate and multivariate logistic regression analyses identified several independent predictors. Specifically, age showed an odds ratio (OR) of 1.05 with a 95% confidence interval (CI) ranging from 1.01 to 1.10 (*P* = 0.013). For sex, the OR was 0.35 (95% CI: 0.15–0.84; *P* = 0.018). Spiculation emerged as a significant predictor with a notably higher OR of 9.49 (95% CI: 3.28–27.46; *P* < 0.001). Additionally, cavity had an OR of 0.31 (95% CI: 0.10-1.00; *P* = 0.049). These findings are summarized in Table 1.

### Screening of the imaging radiomics features

A total of 107 radiomics features were initially extracted. First, nine features with ICC below 0.750 were excluded. The consistency evaluation revealed that the average inter-group and intra-group correlation coefficient values of the remaining 98 imaging radiomics features were in the ranges of 0.761 -1.000 and 0.792-1.000, respectively. This suggests a good inter-observer and intra-observer consistency for these 98 radiomic features. Through correlation analysis screening, 31 plain CT imaging radiomics features and 27 enhanced CT imaging radiomics features were retained. Subsequently, by applying the gradient-boosting decision tree (GBDT) method, 11 plain CT imaging radiomics features and 9 enhanced CT imaging radiomics features were ultimately selected as the optimal features.

### Model development and comparison

**Fig. 4:**
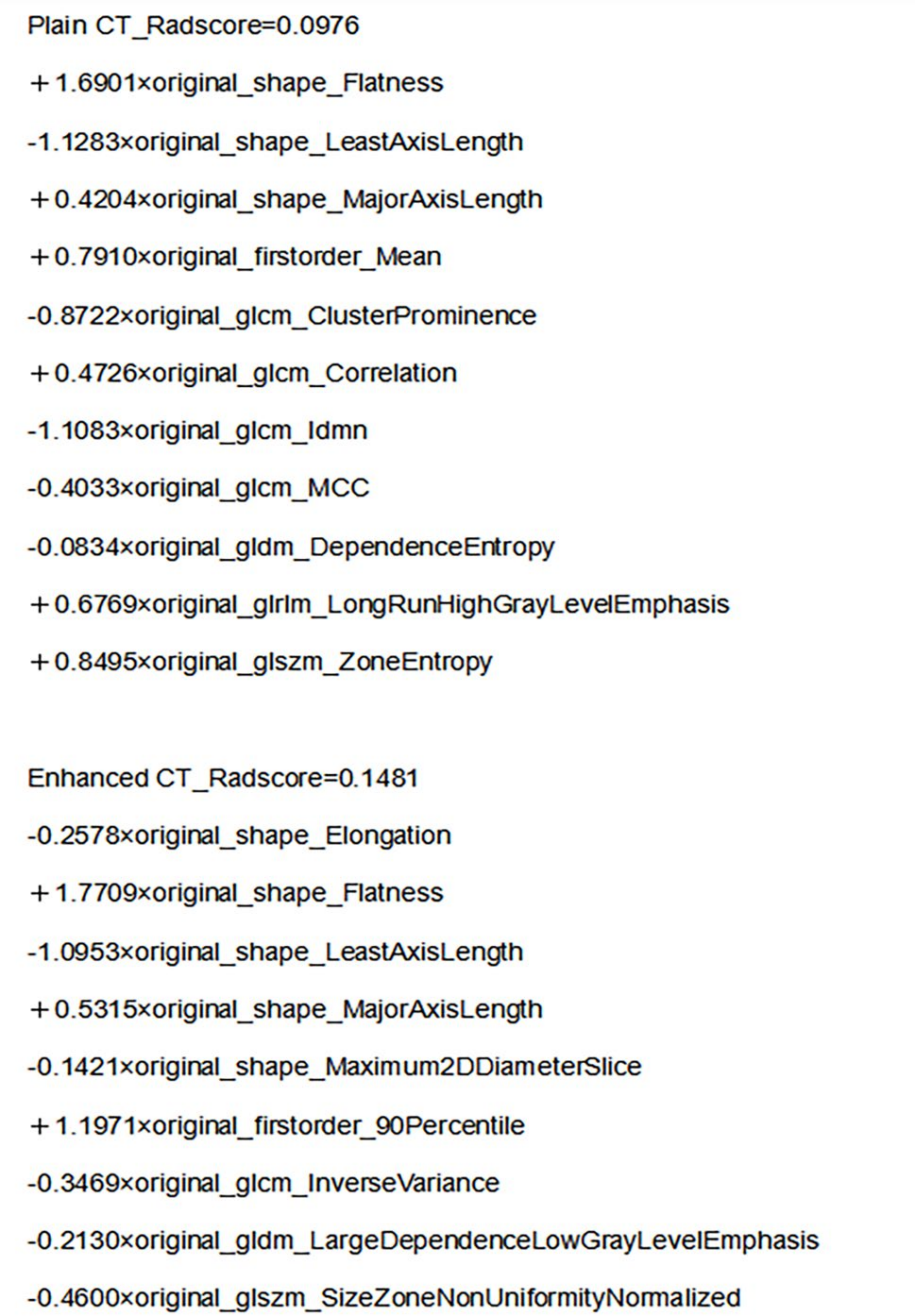
Model formulas for the PRM and the ERM. *PRM* Plain CT radiomics model, *ERM* Enhanced CT radiomics model

In the training dataset, four logistic regression models were developed, namely the CIM, PRM, ERM, and CM. The CIM was established using age, sex, spiculation, and cavity as variables. The model equations for the PRM and ERM are illustrated in Fig. 4. The CM integrated the components of the CIM, PRM, and ERM. As presented in Table 2, the area under the curve (AUC), sensitivity, specificity, and accuracy of these four models were listed. The ERM exhibited higher AUC, sensitivity, specificity, and accuracy values compared to the PRM and CIM. Among all the constructed models, the CM emerged as the optimal one, achieving an AUC of 0.888 in the training dataset and 0.881 in the testing dataset.

**Table 2:** Diagnostic efficacy of the four models in the training and testing datasets

Model	Training dataset				Testing dataset			
	AUC (95% CI)	Sensitivity	Specificity	Accuracy	AUC (95% CI)	Sensitivity	Specificity	Accuracy
CIM	0.795(0.712, 0.877)	0.815	0.678	0.750	0.810(0.695, 0.925)	0.774	0.522	0.667
PRM	0.791(0.712, 0.870)	0.708	0.678	0.694	0.689(0.536, 0.841)	0.548	0.609	0.574
ERM	0.828(0.755, 0.901)	0.831	0.695	0.766	0.864(0.768, 0.960)	0.871	0.696	0.796
CM	0.888(0.830, 0.945)	0.831	0.797	0.815	0.881(0.795, 0.967)	0.806	0.652	0.741

*CIM* the Clinical and image model, *PRM* the Plain CT radiomics model, *ERM* the Enhanced CT radiomics model, *CM* the Combined model, *AUC* Area under the receiver operating characteristic curve, *CI* Confidence interval

The coefficient results of the logistic regression modeling parameters are shown in Supplement Fig. 2. Age, spiculations, C radscore and E radscore represent positive effects. The older the age, the more spiculations exists, the larger the C radscore and E radscore, the greater the probability of malignancy. The presence of male and cavity represents a negative effect, while the presence of male and cavity represents a low probability of malignancy.

**Fig. 5:**
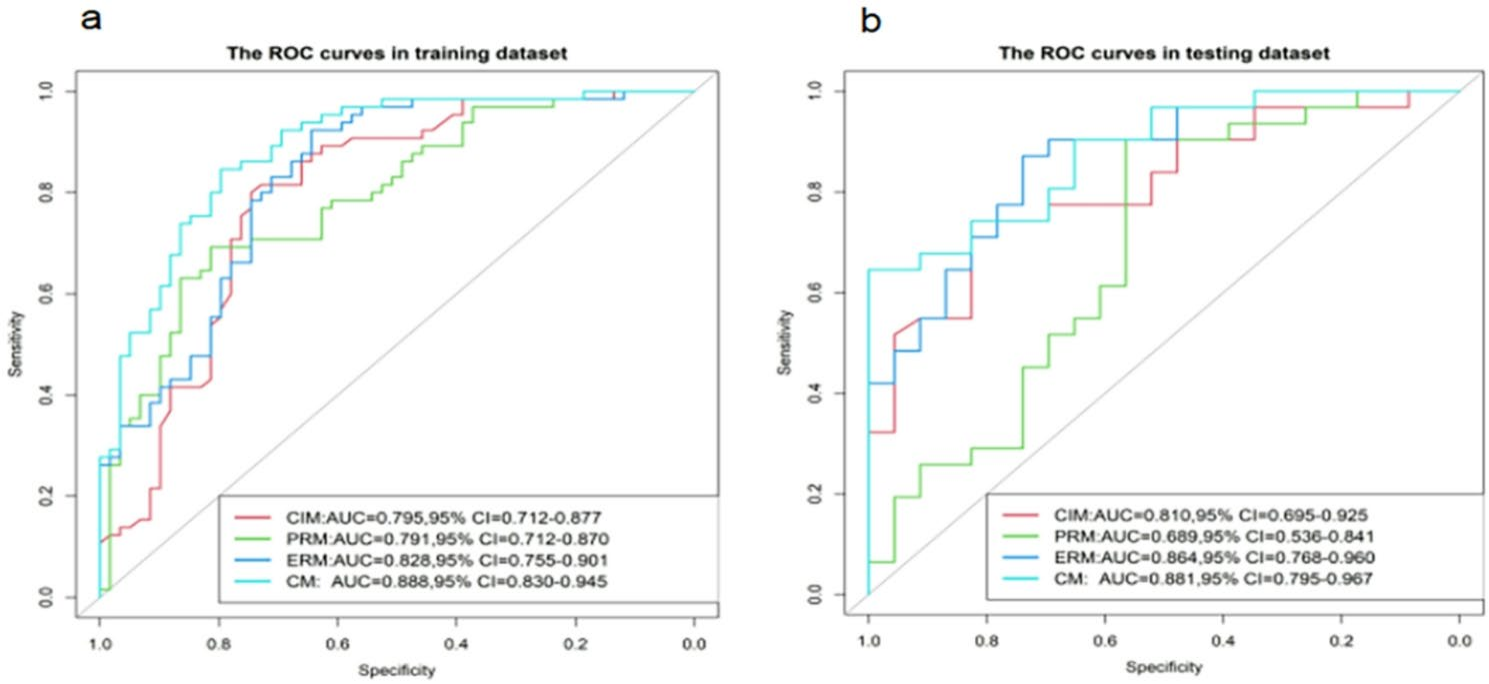
The receiver operating characteristic (ROC) curves for the four models in the training (**a**) and testing (**b**) datasets. *CIM* clinical and image model, *PRM* Plain CT radiomics model, *ERM* Enhanced CT radiomics model, *CM* Combined model, *AUC* Area under the ROC curve, *CI* Confidence interval

**Fig. 6:**
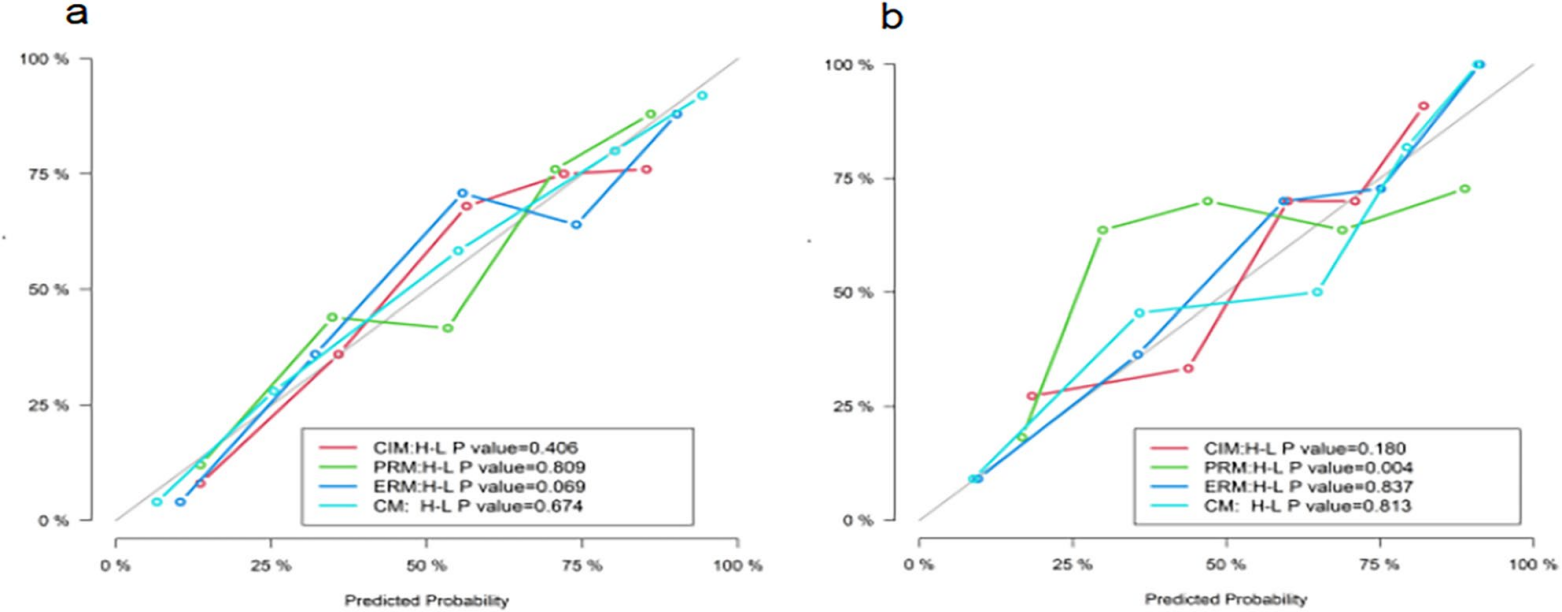
Calibration curves of four models in the training (**a**) and testing (**b**) datasets. The *P* value > 0.050 indicates good fit between the model and ideal model. *CIM* clinical and image model, *PRM* Plain CT radiomics model, *ERM* Enhanced CT radiomics model, *CM* Combined model, *H–L* Hosmer–Lemeshow

Figure 5 illustrates the ROC curves of the four models in both the training and testing datasets, while Fig. 6 depicts their corresponding calibration curves. In the training and testing datasets, the Hosmer-Lemeshow test results for the CIM, ERM, and CM models all exceeded 0.050, suggesting superior calibration capabilities for these models.

The NRI and the IDI were calculated to compare the performance of the different models. In the training dataset, categorical NRI for CM better than PRM (*P* < 0.050). Continuous NRI for CM outperforms CIM, PRM, and ERM (*P* < 0.001). The IDI for CM outperforms CIM, PRM, and ERM(*P* < 0.001). Detailed results are shown in Table 3. In the testing dataset, continuous NRI for CM outperforms CIM, PRM, and ERM(*P* < 0.050). The IDI of the CM outperforms the CIM and the PRM(*P* < 0.050). Detailed results are shown in Table 4.

**Table 3:** Training dataset model performance comparison

Model	Categorical NRI (95% CI)		*P*	Continuous NRI (95% CI)	*P*	IDI(95% CI)	*P*
CIM- PRM		0.108(-0.310–0.095)	0.296	0.140(-0.489–0.209)	0.431	-0.031(-0.141–0.078)	0.575
CIM- ERM		0.032(-0.160–0.224)	0.741	0.294(-0.054–0.642)	0.098	0.058(-0.049–0.165)	0.290
PRM- ERM		0.140(-0.034–0.314)	0.114	0.519(0.179–0.859)	0.003*	0.089(0.018–0.160)	0.014*
CIM- CM		0.134(-0.008–0.276)	0.064	0.947(0.641–1.254)	< 0.001*	0.183(0.113–0.254)	< 0.001*
PRM-CM		0.242(0.077–0.406)	0.004*	1.030(0.728–1.332)	< 0.001*	0.215(0.141–0.289)	< 0.001*
ERM-CM		0.102(-0.030–0.233)	0.129	0.799(0.476–1.122)	< 0.001*	0.126(0.067–0.185)	< 0.001*

*NRI* Net reclassification improvement, *IDI* Integrated discrimination index, *CIM* the Clinical and image model, *PRM* the Plain CT radiomics model, *ERM* the Enhanced CT radiomics model, *CM* the Combined model, *CI* Confidence interval

*P value < 0.050

**Table 4:** Testing dataset model performance comparison

Model	Categorical NRI (95% CI)	*P*	Continuous NRI (95% CI)	*P*	IDI(95% CI)	*P*
CIM- PRM	-0.139(-0.490–0.213)	0.439	-0.292(-0.826–0.242)	0.284	-0.105(-0.284–0.074)	0.250
CIM- ERM	0.271(-0.083–0.624)	0.133	0.485(-0.037–1.007)	0.068	0.122(-0.038–0.281)	0.135
PRM-ERM	0.410(0.159–0.660)	0.001*	1.181(0.779–1.583)	< 0.001*	0.227(0.123–0.330)	< 0.001*
CIM- CM	0.119(-0.158–0.396)	0.399	0.830(0.344–1.317)	0.001*	0.213(0.077–0.349)	0.002*
PRM-CM	0.150(-0.102–0.402)	0.242	0.895(0.419–1.370)	< 0.001*	0.218(0.093–0.343)	0.001*
ERM-CM	0.011(-0.192–0.215)	0.914	0.724(0.220–1.227)	0.005*	0.098(-0.001–0.197)	0.052

*NRI* Net reclassification improvement, *IDI* Integrated discrimination index, *CIM* the Clinical and image model, *PRM* the Plain CT radiomics model, *ERM* the Enhanced CT radiomics model, *CM* the Combined model, *CI* Confidence interval

*P value < 0.050

Supplement Fig. 3 shows the results of the decision curve analysis of the four models in the training set and testing set. Within the broad probability threshold interval, the overall net gain of the ERM model is generally higher than the PRM model, while the CM model showed the largest overall net gain. CM has the highest clinical value in identifying benign lesions and lung adenocarcinoma of Lung-RADS 2022 4B solid lung nodules.

The three domestic / foreign recognized prediction models, one UI AI model, compared the results with the CM with the highest efficacy in this study as shown in Supplement Table 2. The AUC and the accuracy of the CM outperforms the other four models.

## Discussion

In this study, the imaging radiomics model we constructed demonstrated superior diagnostic efficacy over the CIM in distinguishing benign lesions and lung adenocarcinoma of Lung-RADS 2022 category 4B solid lung nodules. ERM has a greater advantage over PRM. Furthermore, the CM is the best model. At the same time, the diagnostic efficiency of CM is also significantly better than that of the three domestic and foreign recognized prediction models and UI AI model. These results all clarify the good clinical application of the CM based on enhanced CT in the differential diagnosis of benign lesions and lung adenocarcinoma of Lung-RADS 2022 category 4B solid lung nodules.

### Conventional imaging features of CT images

Age of onset was higher in the lung adenocarcinoma group when compared with the benign lesion group, this difference may be attributed to decreased immune function, cellular damage induced by long-term chronic diseases, or an increased risk of genetic variation in the older population. Some studies have found that age is an important predictor to distinguishing lung adenocarcinoma of isolated lung nodules from tuberculosis [9, 22, 23]. The studies by Ailing Liu and She et al. pointed to, where age was an independent predictor in the early screening of lung cancer, to distinguish between benign and malignant lung nodules [24, 25]. These are similar to our findings.

The proportion of male patients was less than the proportion of female patients in the lung adenocarcinoma group compared with the benign lesion group. The study by Zhu et al. pointed to, sex as an independent predictor for distinguishing between benign and malignant pulmonary nodules [9]. This is consistent with our study. However, Rui Jing’s study pointed out that there was no significant difference in sex [26]. This may be due to the data offset.

Spiculations were more common in the lung adenocarcinoma group than in the benign lesion group. Several studies have found that spiculation as an important independent predictor for distinguishing the lung adenocarcinoma group and the tuberculosis group in solid nodules [18, 22, 23]. This may be due to the spread of malignant cells within the lung interstitium and intratumoral fibrosis, and the cancer cells invade the surrounding tissues [24]. These results are similar to our findings. Thus, the malignant nodules may have spiculation signs.

The number of cavity in the lung adenocarcinoma group was less than that in the benign lesion group. Zhao’s study identified, cavity as an independent predictor of the lung adenocarcinoma group and the tuberculosis group in solid nodules or masses [18]. These are consistent with our study. This may be because in our study, there were 34 tuberculosis nodules in the benign group, accounting for a large proportion, and the cavity in tuberculosis nodules is one of the more common imaging features; Secondly, 44 inflammatory nodules, which also increases the possibility of cavity presence. However, this study did not refine the features of cavity (wall thickness, satellite focus, etc.), which can be paid attention to in the subsequent work.

### Assessment model performance

The results of this study showed that the diagnostic efficacy of ERM based on enhanced CT was significantly better than PRM. Compared with separate imaging radiomics models and clinical models, the CM has better diagnostic efficacy. A recent study showed that, the combined model (training dataset AUC = 0.951, testing dataset AUC = 0.941) had better diagnostic performance than the imaging radiomics model alone (training dataset AUC = 0.942, testing dataset AUC = 0.934) and the separate clinical model (training dataset AUC = 0.744, testing dataset AUC = 0.698) [27], which is consistent with the results of this study. Other studies have shown that [28], combined model is more conducive to the identification of benign and malignant solid pulmonary nodules than imaging radiomics model and clinical model alone. Compared with the single imaging radiomics model and the clinical model, the comprehensive analysis model integrating multi-source data shows a better efficiency.

### Comparison diagnostic efficacy of the five models in the testing dataset

At present, the academic community has developed a variety of models designed to evaluate the malignant risk of pulmonary nodules, including the Mayo model, the VA model, and the PKUPH model. These models quantify the malignant potential of pulmonary nodules by integrating clinical information with CT imaging features. The Mayo model considered age, cigarettes, cancer, diameter, spiculation, and the upper lobe of the lesion as key factors to predict the malignancy of pulmonary nodules. The VA model is an evaluation model for isolated pulmonary nodules, which estimates the risk of malignancy based on age, nodule diameter, smoke and smoking cessation duration. The PKUPH model integrates age, nodule size, family tumor history, the presence of spiculation sign, calcification status and boundary features, and constructs a statistical prediction model designed to evaluate the malignant probability of pulmonary nodules. Our results showed similarities between the key variables involved in the model in predicting whether pulmonary nodules were malignancy, such as spiculation sign and age as well as sex. In addition, our model incorporates the radiomics properties of plain and enhanced CT radiomics features are of great significance for the identification of benign and malignant pulmonary nodules. Our combined model showed a higher AUC of 0.881, probably due to our inclusion of CT imaging radiomic features.

We recognize that existing models for solid lung nodule diagnosis have a few limitations: traditional clinical models (Mayo, VA, PKUPH) lack quantitative radiomics features and show poor discrimination (AUC 0.410–0.676 in our study). Moreover, few models target the specific subgroup of Lung-RADS 2022 category 4B solid nodules, which require precise stratification to balance diagnostic accuracy and clinical efficiency. Our combined model addresses these gaps by integrating multi-modal data (clinical/imaging + plain/enhanced CT radiomics) and using GBDT for discriminative feature selection, resulting in superior performance (AUC 0.881 in testing dataset) for this subgroup.

Importantly, we acknowledge the potential risk of diagnostic delays associated with model reliance. False-negative predictions could delay treatment for early-stage adenocarcinoma, highlighting that the model should serve as a decision-support tool rather than a standalone diagnostic standard. Clinicians must integrate model predictions with MDT consultation, patient-specific clinical factors, and dynamic follow-up data—especially for nodules with intermediate predicted probabilities—to avoid over-reliance. Prospective multicenter validation and standardized reporting of model outputs will further mitigate this risk, ensuring the model’s safe and effective clinical implementation.

In summary, our combined model improves the differential diagnosis of Lung-RADS 2022 category 4B solid nodules by addressing the limitations of existing models, while careful clinical integration and safeguards can minimize the risk of diagnostic delays.

### The potential heterogeneity in benign lesions

First, to address it, we implemented targeted exclusion criteria: hamartomas were excluded due to their pathognomonic benign features (calcification, fat content, low enhancement) enabling clear identification; tuberculomas with large calcification were not included because they are radiologically definitive on plain CT. Our cohort thus focused on the lesions with ambiguous plain CT findings—cases posing the greatest diagnostic challenges. We propose future studies refine this by exploring the differential diagnosis between specific benign subtypes (e.g., sclerosing pneumocytoma) and lung adenocarcinoma.

### Future research directions

First, future studies could further investigate the differential diagnosis between benign lesions and lung adenocarcinoma presenting as Lung-RADS 2022 category 4 A solid nodules based on multiscale CT radiomics. Second, studies could further explore the differential diagnosis between a specific benign subtype (e.g., sclerosing pneumocytoma) and malignant pulmonary solid nodules. Additionally, the differential diagnosis of pulmonary solid nodules (categories 4 A or 4B) between benign and malignant types could be performed using the delta radiomics method. Finally, future studies could investigate the prognosis and therapeutic efficacy of lung adenocarcinoma.

First, optimizing radiomics methodologies: Future prospective multi-center studies will standardize CT scanning protocols to reduce variability, ensuring radiomics feature reproducibility. Additionally, integrating handcrafted features with deep learning-based radiomics will capture more comprehensive tumor information.

Second, refining prospective data collection: We will collect multi-omics data (biopsy, pathological biomarkers) to construct integrated models, and conduct stratified analysis by nodule subtypes and patient characteristics to develop personalized diagnostic tools. Prospective validation will also incorporate patient-centered outcomes (e.g., treatment response) to verify clinical utility.

Third, expanding radiomics applications: Future research will explore the potential of radiomics features in predicting therapeutic responses(e.g., lung adenocarcinoma), extending their value from differential diagnosis to personalized treatment decision-making.

These directions will address the current study’s single-center and retrospective limitations, enhance the generalizability and clinical translatability of radiomics models, and ultimately improve the management of patients with Lung-RADS 2022 category 4B solid nodules.

### Limitations

There are several limitations to the current study. First, as a retrospective study, there may be inherent biases and limitations related to data collection and patient selection .and prospective studies are needed in the future. Secondly, further research is needed with an external validation group in the future. Third, only one enhanced CT phase was used; more phases should be evaluated in the future. Fourth, only intratumoral radiomics were analyzed—peritumoral information warrants exploration. Finally, despite resampling to reduce slice thickness effects, scanner type differences may persist.

## Conclusion

In conclusion, in this study, we established CIM, PRM, ERM and CM to distinguish between benign lesions and lung adenocarcinoma of Lung-RADS 2022 category 4B solid lung nodules, The CM performed the best in each of these models. In the testing dataset, the CM model was also compared with Mayo model, VA model, PKUPH model and UI AI model, and CM still performed the best among these models.

## Supplementary Information

Supplementary Material 1.

## Data Availability

The datasets generated during and/or analysed during the current study are available from the corresponding author on reasonable request.
