# Supplementary material for: Differential diagnosis of benign lesions and lung adenocarcinoma presenting as lung-RADS 2022 category 4B solid nodules based on multiscale CT radiomics
Source: BMC Cancer. 2026 Mar 22;26:561. doi: 10.1186/s12885-026-15883-w (PMC13137663; doi:10.1186/s12885-026-15883-w)
Supplement: Supplementary file 1 — Supplementary Material 1. [file 12885_2026_15883_MOESM1_ESM.docx]

**Supplement Table 1** Basic information and conventional imaging features about patients in the training and testing datasets

|  | **Training dataset** | | | **Testing dataset** | | |
| --- | --- | --- | --- | --- | --- | --- |
|  | **benign** | **malignant** | ***P*** | **benign** | **malignant** | ***P*** |
|  | ***N***=***59*** | ***N***=***65*** |  | ***N***=***23*** | ***N***=***31*** |  |
| Age | 58.00 [51.50;65.00] | 62.00 [58.00;69.00] | 0.003* | 56.00 [50.00;63.00] | 65.00 [53.00;70.00] | 0.024* |
| Sex |  |  | 0.066 |  |  | 0.085 |
| Famale | 22 (37.30％) | 36 (55.40％) |  | 5 (21.70％) | 15 (48.40％) |  |
| Male | 37 (62.70％) | 29 (44.60％) |  | 18 (78.30％) | 16 (51.60％) |  |
| Smoking status |  |  | 0.254 |  |  | 0.149 |
| Never smoked | 37 (62.70％) | 48 (73.80％) |  | 11 (47.80％) | 22 (71.00％) |  |
| Ex-or current smoker | 22 (37.30％) | 17 (26.20％) |  | 12 (52.20％) | 9 (29.00％) |  |
| Max diameter | 2.50 [2.05;2.85] | 2.40 [2.00;2.80] | 0.438 | 2.78 (±0.61) | 2.46 (±0.50) | 0.046* |
| Mean  diameter | 2.10 [1.75;2.40] | 2.10 [1.80;2.50] | 0.964 | 2.30 [1.95;2.75] | 2.10 [1.80;2.40] | 0.213 |
| Location |  |  | 1.000 |  |  | 0.471 |
| Lower | 23 (39.00％) | 25 (38.50％) |  | 8 (34.80％) | 15 (48.40％) |  |
| Upper and middle | 36 (61.00％) | 40 (61.50％) |  | 15 (65.20％) | 16 (51.60％) |  |
| Spiculation |  |  | <0.001* |  |  | 0.078 |
| Absent | 26 (44.10％) | 6 (9.23％) |  | 7 (30.40％) | 3 (9.68％) |  |
| Present | 33 (55.90％) | 59 (90.80％) |  | 16 (69.60％) | 28 (90.30％) |  |
| Lobulation |  |  | 0.243 |  |  | 0.148 |
| Absent | 15 (25.40％) | 10 (15.40％) |  | 6 (26.10％) | 3 (9.68％) |  |
| Present | 44 (74.60％) | 55 (84.60％) |  | 17 (73.90％) | 28 (90.30％) |  |
| Cavity |  |  | 0.134 |  |  | 0.028* |
| Absent | 47 (79.70％) | 59 (90.80％) |  | 16 (69.60％) | 29 (93.50％) |  |
| Present | 12 (20.30％) | 6 (9.23％) |  | 7 (30.40％) | 2 (6.45％) |  |
| Pleural traction |  |  | 0.830 |  |  | 1.000 |
| Absent | 9 (15.30％) | 8 (12.30％) |  | 3 (13.00％) | 4 (12.90％) |  |
| Present | 50 (84.70％) | 57 (87.70％) |  | 20 (87.00％) | 27 (87.10％) |  |
| Air bronchogram |  |  | 0.915 |  |  | 0.878 |
| Absent | 36 (61.00％) | 38 (58.50％) |  | 12 (52.20％) | 18 (58.10％) |  |
| Present | 23 (39.00％) | 27 (41.50％) |  | 11 (47.80％) | 13 (41.90％) |  |

Categorical variables are expressed as numbers (%); normal distribution continuous variables are expressed as the mean ± standard deviation; abnormal distribution continuous variables are expressed as median (first quartile, third quartile); **P* value < 0.050

**Supplement Table 2** Comparison diagnostic efficacy of the five models in the testing datasets

| model | AUC (95% CI) | Sensitivity | Specificity | Accuracy |
| --- | --- | --- | --- | --- |
| Mayo model | 0.419(0.263-0.575) | 0.645 | 0.435 | 0.574 |
| VA model | 0.410(0.254-0.566) | 1.000 | 0.043 | 0.574 |
| PKUPH model | 0.676(0.494-0.799) | 0.806 | 0.478 | 0.574 |
| UI AI model | 0.675(0.530-0.820) | 0.387 | 0.913 | 0.611 |
| CM | 0.881(0.795- 0.967) | 0.806 | 0.652 | 0.741 |

*Mayo* Mayo Clinic；*VA* Veterans Administration；*PKUPH* Peking University People’s Hospital; *UI AI* United Image Artificial Intelligence; *CM* the combined model

**
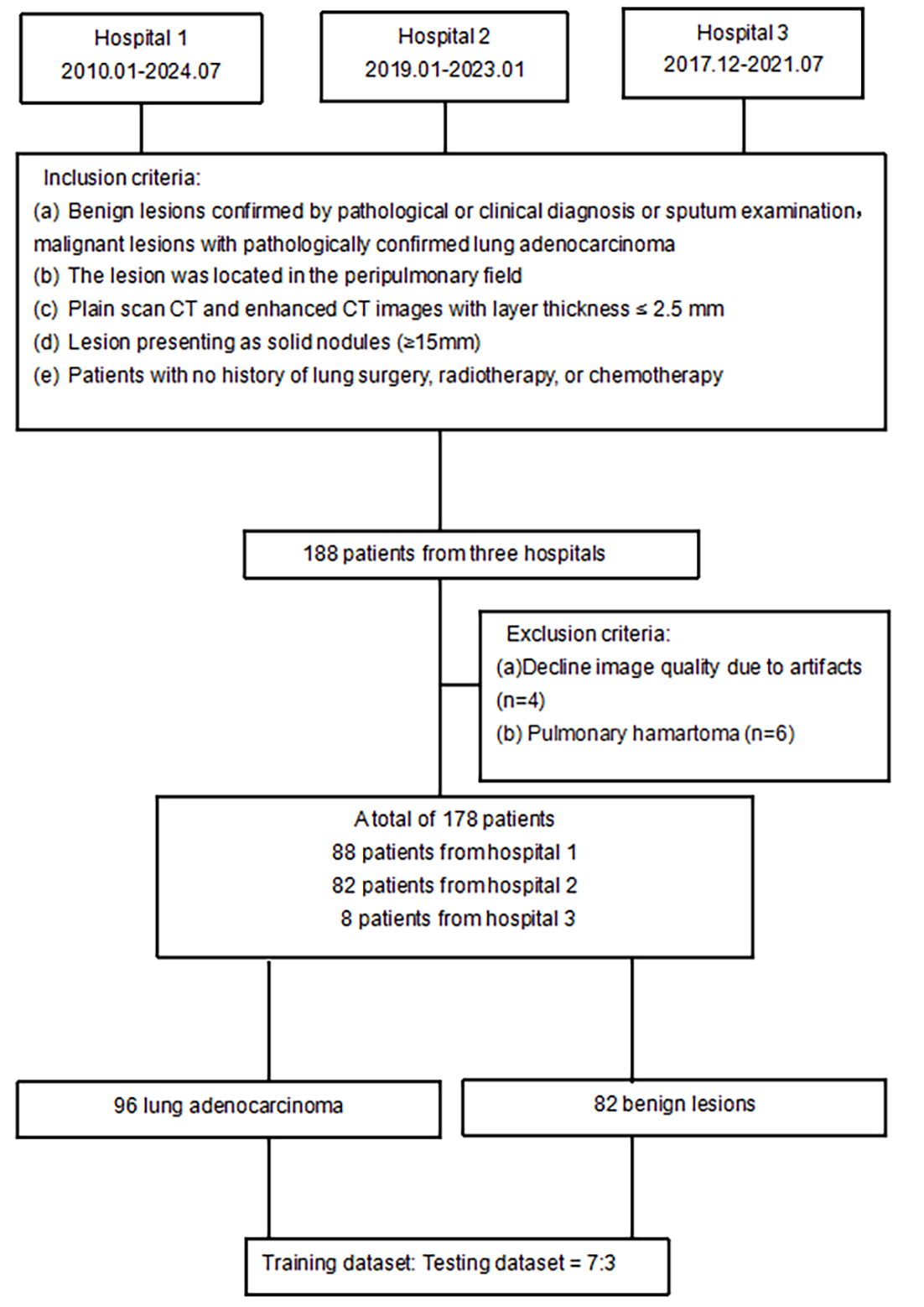
**

**Supplement Fig.1** Flow chart of Lung-RADS 2022 category 4B solid lung nodules selection and grouping


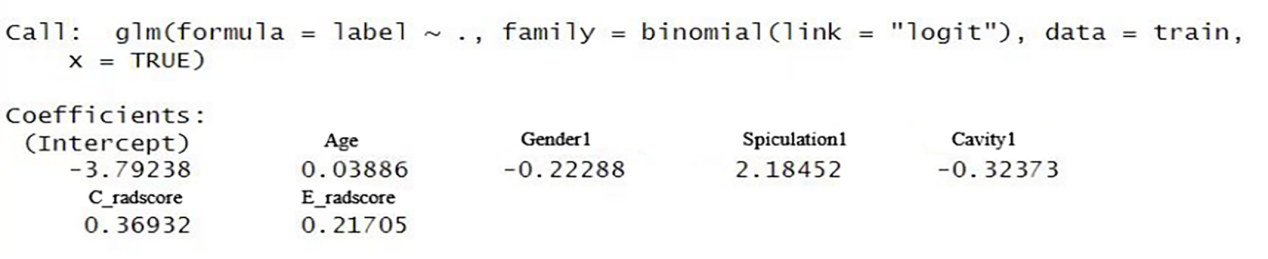


**Supplement Fig.2** Logical regression modeling parameter coefficient


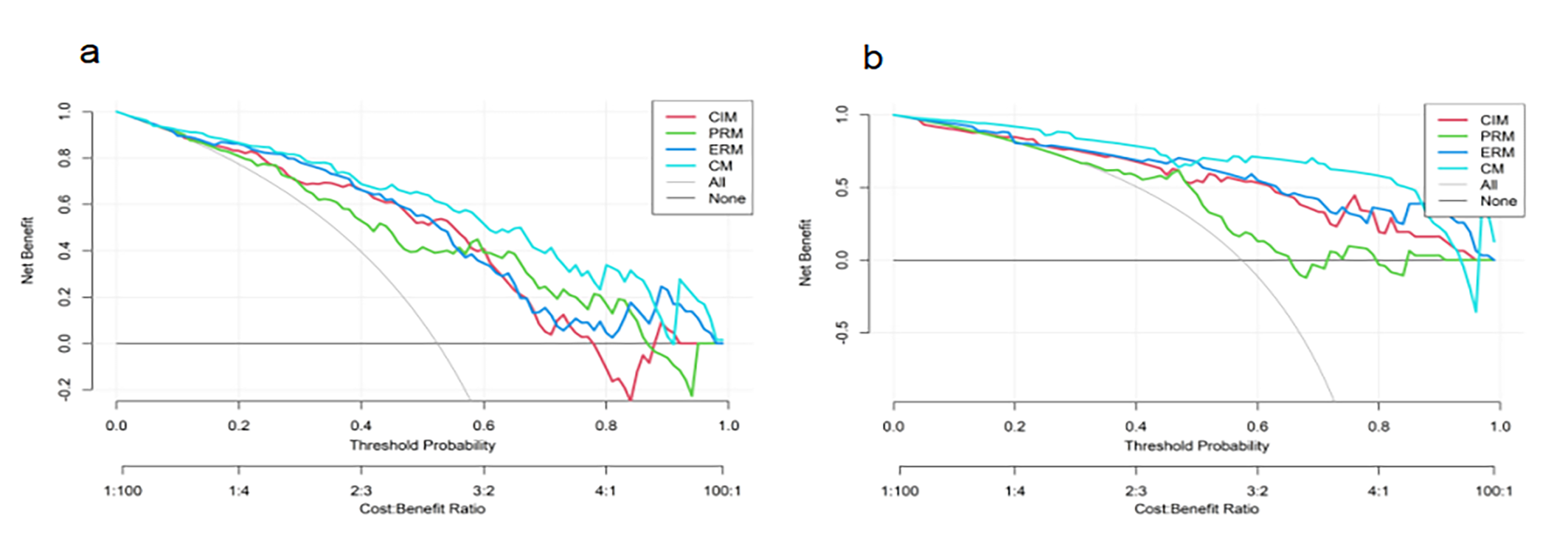


**Supplement Fig.3** The decision curve analysis for four models in the training (**a**) and testing (**b**) datasets.*CIM* clinical and image model, *PRM* plain CT radiomics model, *ERM* enhanced CT radiomics model, *CM* combined model
